# Phase-I trial of survivin inhibition with EZN-3042 in dogs with spontaneous lymphoma

**DOI:** 10.1186/s12917-020-02317-3

**Published:** 2020-03-24

**Authors:** Douglas H. Thamm, Jenette K. Joseph, Barbara J. Rose, Travis K. Meuten, Kristen M. Weishaar

**Affiliations:** 1 Flint Animal Cancer Center, Colorado State University, Fort Collins, CO 80523-1620 USA; 2 Cell and Molecular Biology Graduate Program, Colorado State University, Fort Collins, CO 80523 USA; 3 University of Colorado Cancer Center, Anschutz Medical Campus, Aurora, CO 80045 USA; 4 Department of Microbiology, Immunology and Pathology, College of Veterinary Medicine and Biomedical Sciences, Colorado State University, Fort Collins, CO 80523 USA

**Keywords:** birc5, Canine, Cancer, Antisense, Apoptosis

## Abstract

**Background:**

Lymphoma is a common cancer in dogs. While most dogs receiving chemotherapy experience remission, very few are cured, and median survival times are generally in the 12-month range. Novel approaches to treatment are unquestionably needed. The Inhibitor of Apoptosis Protein (IAP) family member survivin, which is one of the most commonly overexpressed proteins in human cancer, plays a key role in apoptosis resistance, a major cause of drug-resistant treatment failure. Survivin targeting therapies have shown promise preclinically; however, none have been evaluated in dogs to date. The goal of the current study was to determine the safety and pharmacodynamic effects of systemic administration of the anti-survivin locked nucleic acid antisense oligonucleotide EZN-3042 in dogs with lymphoma.

**Results:**

We performed a prospective phase-I clinical trial in dogs with biopsy-accessible peripheral nodal lymphoma. Eighteen dogs were treated with EZN-3042 as a 2-h IV infusion at 5 dose levels, from 3.25 to 8.25 mg/kg twice weekly for 3 treatments. No dose-limiting toxicities were encountered. Reduction in tumor survivin mRNA and protein were observed in 3 of 5 evaluable dogs at the 8.25 mg/kg dose cohort.

**Conclusions:**

In conclusion, reduced survivin expression was demonstrated in lymphoma tissues in the majority of dogs treated with EZN-3042 at 8.25 mg/kg twice weekly, which was associated with minimal adverse effects. This dose may be used in future studies of EZN-3042/chemotherapy combinations in dogs with spontaneous lymphoma and other cancers.

## Background

Non-Hodgkin lymphoma (NHL) is the 7th most common cancer in humans, accounting for approximately 65,000 cases per year [1]. It is the fifth most common, and second fastest growing, cause of human cancer mortality [2]. Likewise, lymphoma is one of the most common cancers encountered in dogs [3]. Both human and canine NHL are characterized by a high likelihood of response to CHOP (cyclophosphamide, doxorubicin, vincristine, prednisone) based chemotherapy; however, 30% of humans and 95% of dogs and will eventually die as a result of drug-resistant relapse, [1, 4–7] demonstrating that new therapeutic approaches are needed.

The dog is a very useful model for the study of human NHL, given remarkable similarities in histology, biology and gene-expression. This includes very similar gross and histological appearance, conserved patterns of organ involvement, comparable prognostic factors, and analogous dysregulation of cell signaling and growth regulation pathways [3, 8–12]. Owing to the relatively compressed time course of disease progression of lymphoma (and cancer in general) in dogs versus humans, the immunocompetence of these patients, and the spontaneous nature of the disease, the study of strategies for chemosensitization can be accomplished expediently in dogs and may more closely mimic the human clinical situation than any other cancer model.

Resistance to apoptosis (programmed cell death) is a hallmark of cancer [13]. The inhibitor of apoptosis proteins (IAP) are a family of conserved inhibitors of cell death with analogs in yeast, vertebrates and invertebrates. Survivin, the product of the *Birc5* gene, is an important anti-apoptotic IAP family member that is unique in that its expression peaks during mitosis, [14] and has a critical role in normal cell division [15]. Although survivin is highly expressed in fetal tissues, expression is nearly undetectable in most terminally differentiated adult cells [15, 16]. Notably, an analysis of 3.5 million transcripts from 19 normal and diseased human tissues identified survivin as one of the most commonly upregulated genes in cancer versus normal tissues [17]. Multiple studies suggest that high survivin expression is an important survival mechanism in cancer cells, and can be associated with inferior clinical outcome in humans and dogs with cancer [18–21]. Importantly, high expression of survivin is a negative prognostic factor in both dogs and humans with lymphoma [18, 20, 22–24]. Survivin also appears to regulate tumor vasculature in a vascular endothelial growth factor-dependent fashion, [25] and may have apoptosis and proliferation-independent roles in tumor cell invasion and metastasis [26]. Survivin is thus an encouraging clinical target.

Several survivin-directed therapeutics have been or are currently undergoing human clinical evaluation. These include the small molecule YM155 (sepantronium bromide), [27–32] and the oligo-based therapeutics LY2181308, [33–37] and EZN-3042, the subject of this study [18, 38–41]. Knockdown of survivin expression using RNA interference, antisense, dominant-negative or pharmacologic approaches has been associated with significant inhibition of proliferation and induction of apoptosis in lymphoma in vitro and in murine xenografts [42–46]. Furthermore, multiple studies have reported enhancement of chemotherapy and rituximab sensitivity in human lymphoma/leukemia cells and xenografts when combined with survivin inhibition [18, 39, 47]; however, trials of survivin inhibition in dogs with neoplasia have yet to be reported in the peer-reviewed literature.

EZN-3042 (Enzon Pharmaceuticals, Piscataway, NJ) is a locked nucleic acid antisense oligonucleotide (LNA-AsODN) that targets and reduces expression of survivin mRNA and protein [38–40]. LNA-AsODNs are single-stranded nucleic acids with locked nucleic acid structures attached, which provides protection against degradation and enhances mRNA binding [48]. EZN-3042 is constructed of 16 nucleic acid monomers; seven of these are replaced with LNAs [40]. Its sequence is **5′-CTCAatccatggCAGc-3′**, with capital letters representing LNAs and lower case letters representing DNA monomers [40]. Importantly, the sequence of EZN-3042 has 100% homology with the canine survivin sequence. EZN-3042 has been shown to down-regulate survivin in two different murine lung cancer xenografts, [38] and in a canine osteosarcoma (OSA) model [21]. EZN-3042 down-regulated survivin in human prostatic carcinoma cells, which induced cell cycle arrest and increased apoptosis and paclitaxel sensitivity both in vitro and in vivo [40]. Furthermore, a phase I clinical trial of EZN-3042 has been completed in humans; treatment was generally well tolerated [41].

We previously evaluated survivin expression in dogs with untreated, World Health Organization stage III-IVa B-cell lymphoma, a population of dogs where few other established prognostic factors exist. A majority of cases expressed survivin protein, and high survivin expression was a negative prognostic factor, as has been observed in humans [20]. We demonstrated similarly that survivin is also commonly expressed in canine OSA tissues, and that elevated expression is associated with a worse clinical outcome [21]. Furthermore, incubation of canine OSA and lymphoma cells with anti-survivin small interfering RNA or EZN-3042 significantly reduced survivin mRNA and protein expression, increased apoptosis, inhibited proliferation, and enhanced chemotherapy sensitivity in canine OSA and lymphoma cells in vitro, [49] and with survivin reduction and increased chemotherapy efficacy in a canine OSA xenograft [21].

The objective of the current study was to evaluate the safety and pharmacodynamic effects of EZN-3042 administration to dogs with spontaneous multicentric lymphoma. This was accomplished through the performance of an open-label, prospective phase-I clinical trial.

## Results

### Patient demographics

Patient information is summarized in Table 1. The median number of prior chemotherapy regimens was 0.5, with a range of 0 to 5. In pre-treated dogs, the median time from last treatment to first EZN-3042 dose was 29 days (range 14–155). Drugs given immediately prior to study enrollment included rabacfosadine (4), doxorubicin (3) and vincristine (1). All pre-treated dogs had documented progressive disease prior to inclusion. Eighteen dogs were enrolled over 5 dose levels (3.25, 4.5, 5.75, 7.0 and 8.25 mg/kg). Seventeen of the 18 dogs completed the planned 3 EZN-3042 infusions: one dog was withdrawn following the first infusion owing to measurable progressive disease (PD).

**Table 1:** Summary of patient population (*n* = 18)

**Age (years)**	Median (range)	8 (4–13)
**Weight (kg)**	Median (range)	33.8 (5.2–43.3)
**Sex**	Male castrated	9 (50%)
	Female spayed	6 (33%)
	Male intact	3 (17%)
**Breed**	Mixed breed	4 (22%)
	Golden retriever	3 (17%)
	Labrador retriever	2 (11%)
	Boxer	2 (11%)
	Other (1 each)	7 (39%)
**Immunophenotype**	B	11 (61%)
	T	6 (33%)
	NA	1 (6%)
**Lines of Previous Treatment**	0	9 (50%)
	1	3 (17%)
	2	3 (17%)
	> 2	3 (17%)

*NA* Not available

### Adverse effects and clinical response

Treatment-related adverse effects (AEs) higher than grade 1 were limited to a single episode of grade 3 thrombocytopenia, which occurred at the 8.25 mg/kg dose. This patient had a history of previous thrombocytopenic episodes which could not be attributed to chemotherapy administration. However, per protocol this dose cohort was expanded to 6 dogs and no additional AEs were noted. No changes in modified ECOG performance status or owner reported quality of life assessments were noted during the study period. A full list of observed AEs is presented in Table 2. The grade 1 AEs noted were relatively equally distributed across EZN-3042 dose cohorts. Seventeen of 18 dogs experienced stable disease (SD) as their best response during the 8-day duration of this trial. PD was noted in 1 patent.

**Table 2:** *Summary of Adverse Events. Numbers refer to the number of study patients experiencing an adverse event of a given grade*

	VCOG-CTCAE v1.1 Grade		
	1	2	3
ALP Elevation	1		
Anemia	2		
AST Elevation	1		
Creatinine Elevation	1		
GGT Elevation	1		
Hypercalcemia	1		
Hypercholesterolemia	1		
Hyperproteinemia	1		
Hypochloremia	2		
Hypokalemia	1		
Hypomagnesemia	2		
Lymphocytosis	1		
Neutrophilia	3		
Monocytosis	1		
Proteinuria	1		
Thrombocytopenia			1

*VCOG-CTCAE v1.1* Veterinary Co-operative Oncology Group Common Terminology Criteria for Adverse Events Following Antineoplastic Therapy v1.1*. ALP* Alkaline phosphatase. *AST* Aspartate aminotransferase, *GGT* Gamma-glutamyltransferase

Seventeen of 18 dogs received additional lymphoma treatment after completion of EZN-3042. The median time from EZN-3042 study completion and initiation of the next lymphoma-specific therapy was 1 day (range 0 to 7). A variety of additional therapies were employed; 16 dogs received cytotoxic chemotherapy and 1 dog was enrolled in another clinical trial. Although not prospectively assessed, retrospective evaluation of AEs associated with the agent/regimen received immediately following EZN-3042 completion suggested an expected incidence of AEs. One dog experienced grade 3 neutropenia and 3 dogs had grade 1–3 elevations in alanine aminotransferase, all following lomustine administration. One dog treated in the 8.25 mg/kg cohort died 7 days following administration of lomustine; however, the dog’s owner did not seek veterinary attention and thus the cause of death (AE versus PD versus other) could not be determined.

### Postmortem findings

Postmortem evaluations were performed in 5 patients at 3 dose levels (3.25, 4.5 and 5.75 mg/kg). In addition to generalized lymphoma in 4 of the 5 patients, other findings reported in more than 1 dog included regional pulmonary fibrosis in 3, lymphoplasmacytic enteritis in 2, and hepatocellular hydropic change in 3. Time of postmortem examination following EZN-3042 treatment initiation ranged from 51 to 443 days, and additional therapy was employed in all patients; thus, attribution of any postmortem findings to EZN-3042 administration is challenging.

### Survivin expression

**Fig. 1:**
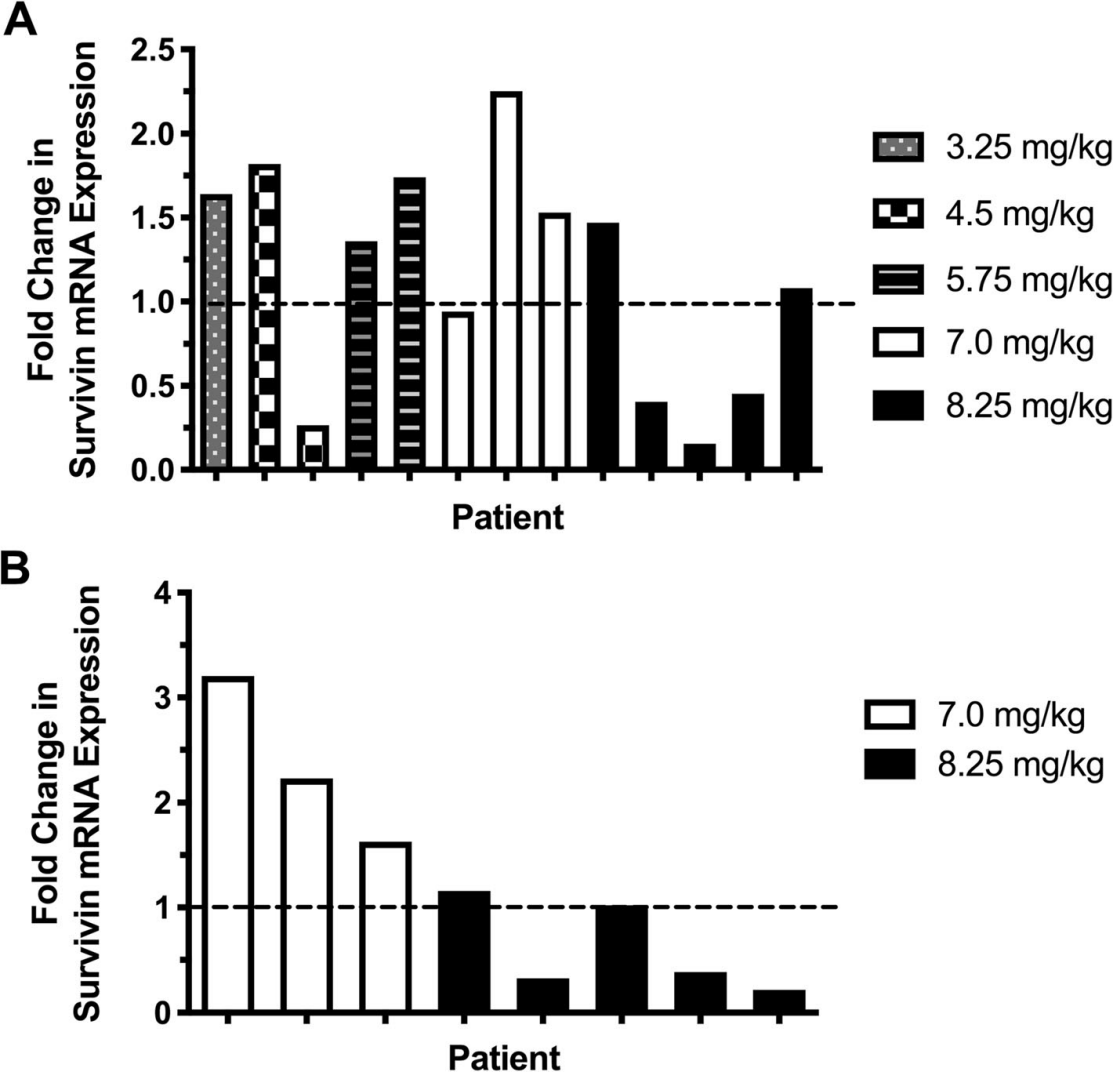
*Changes in relative survivin mRNA expression in tumor tissue and peripheral blood mononuclear cells following EZN-3042 treatment in dogs with spontaneous lymphoma.***a**. Changes in tumor tissue. **b**. Changes in peripheral blood mononuclear cells in dogs treated at the highest 2 dose levels

Pre- and post-treatment mRNA of sufficient quality for quantitative reverse transcriptase polymerase chain reaction (qRT-PCR) was obtained from tumor tissues in 13 of 18 patients. A reduction in relative survivin mRNA expression in tumor tissues was observed in 3 of 5 dogs treated at the highest (8.25 mg/kg) dose cohort (Fig. 1a). Pre-and post-treatment mRNA of sufficient quality for qRT-PCR was obtained from peripheral blood mononuclear cells (PBMC) in 8 of 9 patients in the highest 2 dose cohorts. A reduction in survivin mRNA expression in PBMC was observed in 3 of 5 dogs treated at the highest (8.25 mg/kg) dose cohort (Fig. 1b). Two dogs had parallel reductions in survivin mRNA in both lymph node tissue and PBMC. There was no statistical correlation between changes in lymph node and PBMC (r^2^ = 0.22, *p* = 0.24); however, this analysis was limited by low statistical power.

**Fig. 2:**
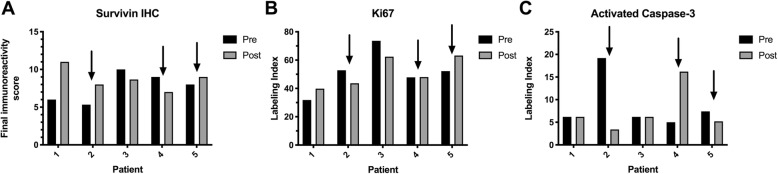
*Changes in survivin expression, proliferation and apoptosis in tumor tissue from dogs with lymphoma treated with EZN-3042.***a** Survivin expression in tumor tissues before and after EZN-3042 treatment as assessed by immunohistochemistry. **b** Tumor cell proliferation assessed immunohistochemically via Ki-67 labeling index in tumor tissues before and after EZN-3042 treatment. **c** Tumor cell apoptosis assessed via activated caspase-3 immunohistochemistry in tumor tissues before and after EZN-3042 treatment. In all figures, black arrows indicate samples where reduction in tumor survivin mRNA expression was observed

Based on the above findings, pathologic interrogation of pre- and post-treatment biopsy tissue was restricted to patients treated in the 8.25 mg/kg dose cohort. Tissue sufficient for immunohistochemistry (IHC) was available in 5 of 6 dogs treated in this cohort. There were no repeatable changes in survivin immunoreactivity (Fig. 2a), Ki67 labeling index (Fig. 2b), or activated caspase-3 activity (Fig. 2c) in these cases. There was likewise no obvious correlation between changes in survivin mRNA expression and changes in these parameters.

## Discussion

Concerns regarding the translatability of many murine tumor models to human cancer patients include differences in immune status, reduced or absent clonal heterogeneity, relative tumor growth rate and tumor burden, tumor location (orthotopic versus heterotopic), differing drug distribution/metabolism, and variations in tolerable drug concentrations. These all may contribute to the observed relatively poor correlation between findings in many rodent studies and early human clinical trials with anticancer agents [50]. More predictive animal models would unquestionably be useful.

Clinical trials in client-owned dogs with spontaneous cancer are potentially informative and, to date, underutilized translational models, as a result of dogs’ comparatively large size, relative outbreeding, immune-competence, and biological/physiological similarity to human cancer patients [51]. Dogs with spontaneous cancer naturally develop therapy resistance as well as recurrence and metastasis. Tumor burdens in canine patients are more similar to humans than their murine counterparts, which may be critical in reference to important cellular and physiological such as clonal heterogeneity and hypoxia [3, 52, 53]. The larger size of canine tumors also facilitates serial tissue sampling and imaging over time, as we have demonstrated in previous studies [12, 54–56]. This is partly due to the fact that chemical restraint is more commonly employed for many procedures, which mitigates owner concerns about patient discomfort.

There is considerable evidence that overexpression of survivin plays a critical role in the outcome in human patients with lymphoma. In multiple studies, high survivin expression has been identified as an independent negative prognostic factor and/or has been associated with an inferior response to chemotherapy [19, 22–24, 57, 58]. Furthermore, knockdown of survivin expression using RNAi, antisense, dominant-negative or pharmacologic approaches has been associated with significant induction of apoptosis and proliferation inhibition in vitro and in murine xenografts [42–46]. Despite this encouraging preclinical activity, and the fact that some objective responses in human NHL patients were observed in early clinical evaluation of the small molecule inhibitor YM155, [28] response rates were disappointingly low in a subsequent single-agent phase-2 investigation [27]. This may have been due to the fact that enrolled patients were heavily pre-treated with large disease burdens, and the conclusions of the investigators were that combinatorial therapy approaches held more promise [27].

In support of combinatorial treatment, multiple studies have reported enhancement of chemotherapy and/or rituximab sensitivity in lymphoma/leukemia cells and xenografts when combined with survivin inhibition [18, 39, 47, 59]. Furthermore, a recent phase-II trial of combined YM155 and rituximab demonstrated durable responses and good tolerability in human patients with relapsed high grade B cell NHL [31]. The goal of the current study was to establish a biologically effective dose of EZN-3042 in lymphoma-bearing dogs, as a precursor to future prospective combinatorial studies.

In this study, EZN-3042 was well tolerated at all doses; a MTD was not reached in dogs with spontaneous lymphoma. A putative biologically effective dose was established at 8.25 mg/kg, based on reduction in survivin mRNA expression in both PBMC and tumor tissue in 3 of 5 dogs treated at this dose level. Surprisingly, there was no correlation between changes in survivin mRNA as assessed by qRT-PCR and changes in protein expression as assessed immunohistochemically. However, the semi-quantitative assessment of survivin expression by IHC may be less sensitive than qRT-PCR, as appears to be the case for several other measured targets in human cancer [60–62]. Despite our previous observations of significant reductions in proliferation and induction of apoptosis in canine lymphoma cells treated with EZN-3042 in vitro, [49] we observed no correlation between alterations in proliferation/apoptosis assessed immunohistochemically and changes in survivin mRNA expression. This could be due to the relatively modest reductions in survivin observed in this trial versus what was observed in vitro, and/or differences in timing between in vitro and clinical assessment. Importantly, EZN-3042 plasma pharmacokinetics were not determined in the present study, so exposure/pharmacodynamic correlations could not be made. In human patients receiving EZN-3042, a terminal plasma half-life of 5.3 h was reported; however, correlations between exposure and biologic effects were not made due to small case numbers (41).

Objective antitumor effects were not observed following EZN-3042 treatment in any dog. This is not unexpected given the very short (8-day) duration of evaluation, and the fact that significant changes in tumor cell proliferation and apoptosis were not observed in tumor biopsies. However, the fact that stable disease was observed in 17 of 18 patients could be indicative of some degree of biologic effect: canine lymphoma is generally a rapidly proliferative disease, with a reported median survival time of only 1 month following diagnosis without treatment.

The ultimate goal of the work reported here was to pave the way for subsequent studies evaluating combinations of survivin inhibition and cytotoxic therapy in canine lymphoma, as a model for the human disease. Although post-EZN-3042 chemotherapy was not standardized, the majority of dogs in this study received cytotoxic chemotherapy soon after the final EZN-3042 dose. There was no obvious potentiation of chemotherapy-associated AEs in those dogs that were pre-treated with EZN-3042, which bodes well for the tolerability of future combinatorial studies. Importantly, probable potentiation of chemotherapy-associated adverse effects was observed in a human study with EZN-3042 [41].

## Conclusions

In conclusion, this is the first study to evaluate the safety and biologic effects of a survivin-inhibiting strategy in dogs with spontaneous cancer, which may represent a very useful model for bridging the observed gap between murine preclinical and human clinical studies. We have defined a safe and biologically effective dose of EZN-3042 which can be employed in future combinatorial tolerability/efficacy trials, as well as defining a suite of pharmacodynamic assays that can be applied to future studies of novel survivin inhibitors.

## Methods

### Study population / entry criteria

This study was conducted with the approval of the Colorado State University Institutional Animal Care and Use Committee and Clinical Review Board. Informed owner consent was obtained prior to study entry. Dogs with biopsy accessible, cytologically confirmed multicentric lymphoma presenting as patients to the Colorado State University Flint Animal Cancer Center were enrolled. Immunophenotype was established via flow cytometry as described [63, 64]. Dogs were free of other complicating concurrent disease conditions as assessed by physical examination and owner history, and needed to weigh at least 5 kg. They needed acceptable laboratory and clinical values to safely undergo therapy; specifically, (1) Total bilirubin not greater than 1.5x the upper limit of normal (ULN); (2) Creatinine not exceeding 2x ULN; (3) At least 2000 neutrophils/uL, 75,000 platelets/uL, hematocrit at least 30%; (4) Modified Eastern Cooperative Oncology Group (ECOG) performance status of 0 or 1 [65]. Pretreatment with chemotherapy was allowed with at least a 14-day washout from their most recent treatment, including corticosteroids. Dogs of any clinical stage were eligible; however, they needed to be substage A for inclusion. Dogs were not re-staged prior to study entry.

### Initial evaluation

Prior to treatment, dogs underwent a complete physical examination and laboratory assessment including body temperature, pulse and respiration; complete blood count, serum biochemistry profile, coagulation profile (prothrombin time, activated partial thromboplastin time) and urinalysis. A cytologically affected peripheral lymph node was biopsied under sedation. One biopsy sample was immediately snap-frozen in liquid nitrogen then stored at -80 °C, and one was placed in formalin for routine histologic processing.

### EZN-3042 treatment

EZN-3042 was generously provided by Enzon Pharmaceuticals (Bridgewater, NJ). Dogs received 3 twice-weekly doses of EZN-3042, administered as a 2-h intravenous infusion. Starting dosage was 3.25 mg/kg, approximately 50% of the single-agent maximum tolerated dose (MTD) as established in humans (Personal Communication, Y. Zhang, Enzon) [66]. Escalation occurred in 1.25 mg/kg dose levels, via a conventional 3 + 3 design. Escalations were performed with 3 dogs per dose level. If a dose-limiting toxicity (DLT) was observed in one dog in a dosing group, then the cohort would be expanded to 6 dogs at that dose level. Escalation to the next higher dose cohort occurred if 0/3 dogs in a cohort experienced a DLT or if only 1/6 dogs in an expanded cohort experienced a DLT. If a DLT attributable to treatment was observed in more than 1 dog at a dose level, then the MTD was considered exceeded, and dose-escalation terminated. The MTD was defined as the highest dose where no more than 1/6 dogs developed a DLT. A total of 5 dose cohorts were planned. Following treatment initiation, dogs were rechecked according to the schedule outlined in Table 3.

**Table 3:** Clinical Trial Evaluation Schedule

	Time Point			
Procedure	Day 0	Day 3–4	Day 7	Day 8
Lymph node biopsy (qRT-PCR, IHC) +/− PBMC collection	X			X
History, physical exam, response assessment	X	X	X	X
CBC, Biochemistry, PT, PTT, Urinalysis	X	X	X	
QOL Questionnaire	X	X	X	X
Body Weight	X	X	X	X
EZN-3042 Administration	X	X	X	

*IHC* Immunohistochemistry. *PBMC* Peripheral blood mononuclear cells, *CBC* Complete blood count. *PT* Prothrombin time. *APTT* Activated thromboplastin time. *QOL* Quality of life

Lymph node biopsies were repeated 1 day following the third EZN-3042 treatment. After this biopsy, dogs were considered off-study and eligible to receive additional treatment as recommended by the veterinarian in charge. In dogs treated at the 2 highest dose cohorts, PBMC were collected at the same time points as lymph node biopsy. Approximately 20 mL of heparinized peripheral blood was collected. PBMC were separated using density gradient centrifugation as described, [7] and cell pellets were snap-frozen and stored at -80 °C until qRT-PCR for survivin mRNA expression.

### Response and adverse effect assessment

Clinical responses were assessed according to the Veterinary Cooperative Oncology Group Response Evaluation Criteria for Peripheral Nodal Lymphoma in Dogs (v1.0), [67] and classified as complete response (CR), partial response (PR), SD, or PD.

Adverse effects (AEs) were graded according to the Veterinary Co-operative Oncology Group Common Terminology Criteria for Adverse Events Following Antineoplastic Therapy v1.1 [65]. A dose-limiting toxicity (DLT) was defined as a Grade 3 or higher toxicity in any category, except neutropenia, where asymptomatic grade 3 toxicity was not considered dose-limiting. A Quality of life questionnaire was completed by the owner at the time of each visit, and a modified ECOG performance status assigned.

### Survivin expression

Expression of survivin mRNA was assessed in pre and post-EZN-3042 snap-frozen biopsy samples and PBMC utilizing qRT-PCR and IHC. qRT-PCR was performed essentially as we have previously described [21]. Briefly, RNA was extracted using the Qiashredder and RNeasy Kit (Qiagen, Chatsworth, CA) and quality assessed using a NanoDrop spectrophotometer (ThermoFisher, Waltham, MA). RNA was reverse-transcribed using the Omniscript® RT Kit (Qiagen). SYBR Green PCR reactions using canine survivin and HPRT-specific primers were performed in duplicate on an Mx3000P™ thermal cycler (Stratagene, San Diego, CA). Average threshold (Ct) values were calculated to evaluate changes in survivin gene expression relative to HPRT using the Relative Expression Software Tool (REST) v2.0.13 (Qiagen).

Expression of survivin protein was assessed semi-quantitatively in formalin fixed paraffin embedded lymphoma biopsy samples utilizing IHC as we have described, [20, 21] with a validated polyclonal rabbit anti-human survivin antibody (Novus Biologicals, Centennial, CO), utilizing an automated stainer (Discovery, Ventana Systems, Harvard, MA). Paraffin sections of normal canine lymph node served as both positive and negative controls, and omission of the primary antibody was as a secondary negative control in each run. Both nuclear and cytoplasmic survivin immunoreactivity were scored by 1 individual (TYM), who was blinded to patient identity and biopsy timing. Briefly, the proportion of cells demonstrating positive immunoreactivity in each sample was scored according to a semi-quantitative approach where tumors were grouped into 5 classes (0 = 0%, 1 = 1–10%, 2 = 10–25%, 3 = 25–50%, 4 > 50%). The intensity of survivin staining (0 = negative, 1 = weak, 2 = moderate, 3 = strong, 4 = intense) was also assessed. A final immunoreactivity score was then determined by multiplying the percentage score by the intensity grade, with a range of possible scores of 0–16.

### Proliferation and apoptosis assessment

Tumor cell proliferation and apoptosis was assessed in pre- and post-EZN-3042 samples in a blinded fashion by one investigator (TYM) utilizing IHC for Ki-67 and activated caspase-3, as we have described previously in canine tumor tissues [55, 68]. Images were obtained using an Axioplan 2 microscope coupled with an AxioCam HRc camera (Zeiss, Oberkochen, Germany) and results were determine by enumerating the number of Ki67-positive nuclei or activated caspase-3 positive cells per 20x field in 7 random fields per tissue section.

### Statistical analysis

Descriptive statistics were used to summarize demographic information and AEs. Correlation between changes in survivin expression in paired biopsy and PBMC samples was assessed using linear regression. Statistical analysis was carried out using commercial software (Prism 8, GraphPad Software, La Jolla, CA).

## Data Availability

The datasets used and/or analyzed during the current study are available from the corresponding author upon reasonable request.

## Abbreviations

AEs: Adverse events
CHOP: Cyclophosphamide-doxorubicin-vincristine-prednisone
DLT: Dose-limiting toxicity
ECOG: Eastern Cooperative Oncology Group
IAP: Inhibitor of apoptosis proteins
IHC: Immunohistochemistry
LNA-AsODN: Locked nucleic acid antisense oligonucleotide
MTD: Maximum tolerated dose
NHL: Non Hodgkin lymphoma
OSA: Osteosarcoma
PBMC: Peripheral blood mononuclear cells
PD: Progressive disease
qRT-PCR: Quantitative reverse-transcriptase polymerase chain reaction
SD: Stable disease
ULN: Upper limit of normal

## References

[1] Leukemia and Lymphoma Society. Facts, 2010-2011. White Plains: Leukemia and Lymphoma Society; 2011.

[2] Jemal A, Tiwari RC, Murray T, Ghafoor A, Samuels A, Ward E, Feuer EJ, Thun MJ (2004). Cancer statistics, 2004. CA Cancer J Clin.

[3] Vail DM, Thamm DH. Spontaneously occurring tumors in companion animals as models for drug development. In: Teicher BA, Andrews PA, editors. Anticancer Drug Development Guide: Preclinical Screening, Clinical Trials, and Approval (2nd Ed). Totowa: Humana Press; 2004. p. 259–84.

[4] Garrett LD, Thamm DH, Chun R, Dudley R, Vail DM (2002). Evaluation of a 6-month chemotherapy protocol with no maintenance therapy for dogs with lymphoma. J Vet Intern Med.

[5] Hosoya K, Kisseberth WC, Lord LK, Alvarez FJ, Lara-Garcia A, Kosarek CE, London CA, Couto CG (2007). Comparison of COAP and UW-19 protocols for dogs with multicentric lymphoma. J Vet Intern Med.

[6] MacDonald VS, Thamm DH, Kurzman ID, Turek MM, Vail DM (2005). Does L-asparaginase influence efficacy or toxicity when added to a standard CHOP protocol for dogs with lymphoma?. J Vet Intern Med.

[7] Turek MM, Thamm DH, Mitzey A, Kurzman ID, Huelsmeyer MK, Dubielzig RR, Vail DM (2007). Human granulocyte-macrophage colony-stimulating factor DNA cationic-lipid complexed autologous tumour cell vaccination in the treatment of canine B-cell multicentric lymphoma. Vet Comp Oncol.

[8] Valli VE, San Myint M, Barthel A, Bienzle D, Caswell J, Colbatzky F, Durham A, Ehrhart EJ, Johnson Y, Jones C (2011). Classification of canine malignant lymphomas according to the World Health Organization criteria. Vet Pathol.

[9] Breen M, Modiano JF (2008). Evolutionarily conserved cytogenetic changes in hematological malignancies of dogs and humans--man and his best friend share more than companionship. Chromosom Res.

[10] Fosmire SP, Thomas R, Jubala CM, Wojcieszyn JW, Valli VE, Getzy DM, Smith TL, Gardner LA, Ritt MG, Bell JS (2007). Inactivation of the p16 cyclin-dependent kinase inhibitor in high-grade canine non-Hodgkin's T-cell lymphoma. Vet Pathol.

[11] Thomas R, Seiser EL, Motsinger-Reif A, Borst L, Valli VE, Kelley K, Suter SE, Argyle D, Burgess K, Bell J (2011). Refining tumor-associated aneuploidy through 'genomic recoding' of recurrent DNA copy number aberrations in 150 canine non-Hodgkin lymphomas. Leuk Lymphoma.

[12] Honigberg LA, Smith AM, Sirisawad M, Verner E, Loury D, Chang B, Li S, Pan Z, Thamm DH, Miller RA (2010). The Bruton tyrosine kinase inhibitor PCI-32765 blocks B-cell activation and is efficacious in models of autoimmune disease and B-cell malignancy. Proc Natl Acad Sci U S A.

[13] Hanahan D, Weinberg RA (2000). The hallmarks of cancer. Cell.

[14] Li F, Ambrosini G, Chu EY, Plescia J, Tognin S, Marchisio PC, Altieri DC (1998). Control of apoptosis and mitotic spindle checkpoint by survivin. Nature.

[15] Altieri DC (2003). Survivin and apoptosis control. Adv Cancer Res.

[16] Ambrosini G, Adida C, Altieri DC (1997). A novel anti-apoptosis gene, survivin, expressed in cancer and lymphoma. Nat Med.

[17] Velculescu VE, Madden SL, Zhang L, Lash AE, Yu J, Rago C, Lal A, Wang CJ, Beaudry GA, Ciriello KM (1999). Analysis of human transcriptomes. Nat Genet.

[18] Morrison DJ, Hogan LE, Condos G, Bhatla T, Germino N, Moskowitz NP, Lee L, Bhojwani D, Horton TM, Belitskaya-Levy I (2012). Endogenous knockdown of survivin improves chemotherapeutic response in ALL models. Leukemia.

[19] Zyada MM (2011). Relationship of survivin to clinical drug resistance in Burkitt's lymphoma of the head and neck region. Med Oncol.

[20] Rebhun RB, Lana SE, Ehrhart EJ, Charles JB, Thamm DH (2008). Comparative analysis of survivin expression in untreated and relapsed canine lymphoma. J Vet Intern Med.

[21] Shoeneman JK, Ehrhart EJ, Eickhoff JC, Charles JB, Powers BE, Thamm DH (2012). Expression and function of survivin in canine osteosarcoma. Cancer Res.

[22] Adida C, Haioun C, Gaulard P, Lepage E, Morel P, Briere J, Dombret H, Reyes F, Diebold J, Gisselbrecht C (2000). Prognostic significance of survivin expression in diffuse large B-cell lymphomas. Blood.

[23] Markovic O, Marisavljevic D, Cemerikic V, Perunicic M, Savic S, Filipovic B, Mihaljevic B (2011). Clinical and prognostic significance of apoptotic profile in patients with newly diagnosed nodal diffuse large B-cell lymphoma (DLBCL). Eur J Haematol.

[24] Schlette EJ, Medeiros LJ, Goy A, Lai R, Rassidakis GZ (2004). Survivin expression predicts poorer prognosis in anaplastic large-cell lymphoma. J Clin Oncol.

[25] Tran J, Master Z, Yu JL, Rak J, Dumont DJ, Kerbel RS (2002). A role for survivin in chemoresistance of endothelial cells mediated by VEGF. Proc Natl Acad Sci U S A.

[26] Mehrotra S, Languino LR, Raskett CM, Mercurio AM, Dohi T, Altieri DC (2010). IAP regulation of metastasis. Cancer Cell.

[27] Cheson BD, Bartlett NL, Vose JM, Lopez-Hernandez A, Seiz AL, Keating AT, Shamsili S (2012). A phase II study of the survivin suppressant YM155 in patients with refractory diffuse large B-cell lymphoma. Cancer.

[28] Tolcher AW, Mita A, Lewis LD, Garrett CR, Till E, Daud AI, Patnaik A, Papadopoulos K, Takimoto C, Bartels P (2008). Phase I and pharmacokinetic study of YM155, a small-molecule inhibitor of survivin. J Clin Oncol.

[29] Clemens MR, Gladkov OA, Gartner E, Vladimirov V, Crown J, Steinberg J, Jie F, Keating A (2015). Phase II, multicenter, open-label, randomized study of YM155 plus docetaxel as first-line treatment in patients with HER2-negative metastatic breast cancer. Breast Cancer Res Treat.

[30] Kelly RJ, Thomas A, Rajan A, Chun G, Lopez-Chavez A, Szabo E, Spencer S, Carter CA, Guha U, Khozin S (2013). A phase I/II study of sepantronium bromide (YM155, survivin suppressor) with paclitaxel and carboplatin in patients with advanced non-small-cell lung cancer. Ann Oncol.

[31] Papadopoulos KP, Lopez-Jimenez J, Smith SE, Steinberg J, Keating A, Sasse C, Jie F, Thyss A (2016). A multicenter phase II study of sepantronium bromide (YM155) plus rituximab in patients with relapsed aggressive B-cell non-Hodgkin lymphoma. Leuk Lymphoma.

[32] Kudchadkar R, Ernst S, Chmielowski B, Redman BG, Steinberg J, Keating A, Jie F, Chen C, Gonzalez R, Weber J (2015). A phase 2, multicenter, open-label study of sepantronium bromide (YM155) plus docetaxel in patients with stage III (unresectable) or stage IV melanoma. Cancer Med.

[33] Tanioka M, Nokihara H, Yamamoto N, Yamada Y, Yamada K, Goto Y, Fujimoto T, Sekiguchi R, Uenaka K, Callies S (2011). Phase I study of LY2181308, an antisense oligonucleotide against survivin, in patients with advanced solid tumors. Cancer Chemother Pharmacol.

[34] Talbot DC, Ranson M, Davies J, Lahn M, Callies S, Andre V, Kadam S, Burgess M, Slapak C, Olsen AL (2010). Tumor survivin is downregulated by the antisense oligonucleotide LY2181308: a proof-of-concept, first-in-human dose study. Clin Cancer Res.

[35] Erba HP, Sayar H, Juckett M, Lahn M, Andre V, Callies S, Schmidt S, Kadam S, Brandt JT, Van Bockstaele D (2013). Safety and pharmacokinetics of the antisense oligonucleotide (ASO) LY2181308 as a single-agent or in combination with idarubicin and cytarabine in patients with refractory or relapsed acute myeloid leukemia (AML). Investig New Drugs.

[36] Natale R, Blackhall F, Kowalski D, Ramlau R, Bepler G, Grossi F, Lerchenmuller C, Pinder-Schenck M, Mezger J, Danson S (2014). Evaluation of antitumor activity using change in tumor size of the survivin antisense oligonucleotide LY2181308 in combination with docetaxel for second-line treatment of patients with non-small-cell lung cancer: a randomized open-label phase II study. J Thorac Oncol.

[37] Wiechno P, Somer BG, Mellado B, Chlosta PL, Cervera Grau JM, Castellano D, Reuter C, Stockle M, Kamradt J, Pikiel J (2014). A randomised phase 2 study combining LY2181308 sodium (survivin antisense oligonucleotide) with first-line docetaxel/prednisone in patients with castration-resistant prostate cancer. Eur Urol.

[38] Sapra P, Wang M, Bandaru R, Zhao H, Greenberger LM, Horak ID (2010). Down-modulation of survivin expression and inhibition of tumor growth in vivo by EZN-3042, a locked nucleic acid antisense oligonucleotide. Nucleosides Nucleotides Nucleic Acids.

[39] Park E, Gang EJ, Hsieh YT, Schaefer P, Chae S, Klemm L, Huantes S, Loh M, Conway EM, Kang ES (2011). Targeting survivin overcomes drug resistance in acute lymphoblastic leukemia. Blood.

[40] Hansen JB, Fisker N, Westergaard M, Kjaerulff LS, Hansen HF, Thrue CA, Rosenbohm C, Wissenbach M, Orum H, Koch T (2008). SPC3042: a proapoptotic survivin inhibitor. Mol Cancer Ther.

[41] Raetz EA, Morrison D, Romanos-Sirakis E, Gaynon P, Sposto R, Bhojwani D, Bostrom BC, Brown P, Eckroth E, Cassar J (2014). A phase I study of EZN-3042, a novel survivin messenger ribonucleic acid (mRNA) antagonist, administered in combination with chemotherapy in children with relapsed acute lymphoblastic leukemia (ALL): a report from the therapeutic advances in childhood leukemia and lymphoma (TACL) consortium. J Pediatr Hematol Oncol.

[42] Ansell SM, Arendt BK, Grote DM, Jelinek DF, Novak AJ, Wellik LE, Remstein ED, Bennett CF, Fielding A (2004). Inhibition of survivin expression suppresses the growth of aggressive non-Hodgkin's lymphoma. Leukemia.

[43] Congmin G, Mu Z, Yihui M, Hanliang L (2006). Survivin--an attractive target for RNAi in non-Hodgkin's lymphoma, Daudi cell line as a model. Leuk Lymphoma.

[44] Gu CM, Zhu YK, Ma YH, Zhang M, Liao B, Wu HY, Lin HL (2006). Knockdown of survivin gene by vector-based short hairpin RNA technique induces apoptosis and growth inhibition in Burkitt's lymphoma Raji cell line. Neoplasma.

[45] Kanwar JR, Shen WP, Kanwar RK, Berg RW, Krissansen GW (2001). Effects of survivin antagonists on growth of established tumors and B7-1 immunogene therapy. J Natl Cancer Inst.

[46] Kita A, Nakahara T, Yamanaka K, Nakano K, Nakata M, Mori M, Kaneko N, Koutoku H, Izumisawa N, Sasamata M (2011). Antitumor effects of YM155, a novel survivin suppressant, against human aggressive non-Hodgkin lymphoma. Leuk Res.

[47] Kita A, Mitsuoka K, Kaneko N, Nakata M, Miyoshi S, Jitsuoka M, Yamanaka K, Noda A, Mori M, Nakahara T (2012). Sepantronium bromide (YM155) enhances response of human B-cell non-Hodgkin lymphoma to rituximab. J Pharmacol Exp Ther.

[48] Kurreck J, Wyszko E, Gillen C, Erdmann VA (2002). Design of antisense oligonucleotides stabilized by locked nucleic acids. Nucleic Acids Res.

[49] Shoeneman JK, Ehrhart EJ, Charles JB, Thamm DH (2016). Survivin inhibition via EZN-3042 in canine lymphoma and osteosarcoma. Vet Comp Oncol.

[50] Johnson JI, Decker S, Zaharevitz D, Rubinstein LV, Venditti JM, Schepartz S, Kalyandrug S, Christian M, Arbuck S, Hollingshead M (2001). Relationships between drug activity in NCI preclinical in vitro and in vivo models and early clinical trials. Br J Cancer.

[51] LeBlanc AK, Breen M, Choyke P, Dewhirst M, Fan TM, Gustafson DL, Helman LJ, Kastan MB, Knapp DW, Levin WJ (2016). Perspectives from man's best friend: National Academy of Medicine's Workshop on Comparative Oncology. Sci Transl Med.

[52] Paoloni M, Khanna C (2008). Translation of new cancer treatments from pet dogs to humans. Nat Rev Cancer.

[53] Khanna C, Lindblad-Toh K, Vail D, London C, Bergman P, Barber L, Breen M, Kitchell B, McNeil E, Modiano JF (2006). The dog as a cancer model. Nat Biotechnol.

[54] Vail DM, Thamm DH, Reiser H, Ray AS, Wolfgang GH, Watkins WJ, Babusis D, Henne IN, Hawkins MJ, Kurzman ID (2009). Assessment of GS-9219 in a pet dog model of non-Hodgkin's lymphoma. Clin Cancer Res.

[55] Thamm DH, Kurzman ID, Clark MA, Ehrhart EJ, Kraft SL, Gustafson DL, Vail DM (2010). Preclinical investigation of PEGylated tumor necrosis factor alpha in dogs with spontaneous tumors: phase I evaluation. Clin Cancer Res.

[56] Thamm DH, Kurzman ID, King I, Li Z, Sznol M, Dubielzig RR, Vail DM, MacEwen EG (2005). Systemic administration of an attenuated, tumor-targeting Salmonella typhimurium to dogs with spontaneous neoplasia: phase I evaluation. Clin Cancer Res.

[57] Martinez A, Bellosillo B, Bosch F, Ferrer A, Marce S, Villamor N, Ott G, Montserrat E, Campo E, Colomer D (2004). Nuclear survivin expression in mantle cell lymphoma is associated with cell proliferation and survival. Am J Pathol.

[58] Watanuki-Miyauchi R, Kojima Y, Tsurumi H, Hara T, Goto N, Kasahara S, Saio M, Moriwaki H, Takami T (2005). Expression of survivin and of antigen detected by a novel monoclonal antibody, T332, is associated with outcome of diffuse large B-cell lymphoma and its subtypes. Pathol Int.

[59] Sharma H, Sen S, Lo Muzio L, Mariggio A, Singh N (2005). Antisense-mediated downregulation of anti-apoptotic proteins induces apoptosis and sensitizes head and neck squamous cell carcinoma cells to chemotherapy. Cancer Biol Ther.

[60] Rosa FE, Silveira SM, Silveira CG, Bergamo NA, Neto FA, Domingues MA, Soares FA, Caldeira JR, Rogatto SR (2009). Quantitative real-time RT-PCR and chromogenic in situ hybridization: precise methods to detect HER-2 status in breast carcinoma. BMC Cancer.

[61] Sinn HP, Schneeweiss A, Keller M, Schlombs K, Laible M, Seitz J, Lakis S, Veltrup E, Altevogt P, Eidt S (2017). Comparison of immunohistochemistry with PCR for assessment of ER, PR, and Ki-67 and prediction of pathological complete response in breast cancer. BMC Cancer.

[62] Wallander ML, Geiersbach KB, Tripp SR, Layfield LJ (2012). Comparison of reverse transcription-polymerase chain reaction, immunohistochemistry, and fluorescence in situ hybridization methodologies for detection of echinoderm microtubule-associated proteinlike 4-anaplastic lymphoma kinase fusion-positive non-small cell lung carcinoma: implications for optimal clinical testing. Arch Pathol Lab Med.

[63] Wilkerson MJ, Dolce K, Koopman T, Shuman W, Chun R, Garrett L, Barber L, Avery A (2005). Lineage differentiation of canine lymphoma/leukemias and aberrant expression of CD molecules. Vet Immunol Immunopathol.

[64] Rout ED, Avery PR (2017). Lymphoid neoplasia: correlations between morphology and flow cytometry. Vet Clin North Am Small Anim Pract.

[65] Veterinary Cooperative Oncology Group - common terminology criteria for adverse events (VCOG-CTCAE) following chemotherapy or biological antineoplastic therapy in dogs and cats v1.1. Vet Comp Oncol. 2016;14(4):417–46. 10.1111/vco.283 28530307

[66] Tolcher AW, Quinn DI, Ferrari A, Ahmann F, Giaccone G, Drake T, Keating A, de Bono JS (2012). A phase II study of YM155, a novel small-molecule suppressor of survivin, in castration-resistant taxane-pretreated prostate cancer. Ann Oncol.

[67] Vail DM, Michels GM, Khanna C, Selting KA, London CA (2010). Response evaluation criteria for peripheral nodal lymphoma in dogs (v1.0)--a veterinary cooperative oncology group (VCOG) consensus document. Vet Comp Oncol.

[68] Wittenburg LA, Bisson L, Rose BJ, Korch C, Thamm DH (2011). The histone deacetylase inhibitor valproic acid sensitizes human and canine osteosarcoma to doxorubicin. Cancer Chemother Pharmacol.

